# A new look at effective interactions between microgel particles

**DOI:** 10.1038/s41467-018-07332-5

**Published:** 2018-11-28

**Authors:** Maxime J. Bergman, Nicoletta Gnan, Marc Obiols-Rabasa, Janne-Mieke Meijer, Lorenzo Rovigatti, Emanuela Zaccarelli, Peter Schurtenberger

**Affiliations:** 1 Division of Physical Chemistry, Department of Chemistry, Lund University, PO Box 124, SE-22100 Lund, Sweden; 2 CNR-ISC and Department of Physics, Sapienza University of Rome, Piazzale A. Moro 2, 00185 Roma, Italy; 3 Present Address: CR Competence AB, Naturvetarevägen 14, 22362 Lund, Sweden; 4 Present Address: Department of Physics, University of Konstanz, PO Box 688, D-78457 Konstanz, Germany

## Abstract

Thermoresponsive microgels find widespread use as colloidal model systems, because their temperature-dependent size allows facile tuning of their volume fraction in situ. However, an interaction potential unifying their behavior across the entire phase diagram is sorely lacking. Here we investigate microgel suspensions in the fluid regime at different volume fractions and temperatures, and in the presence of another population of small microgels, combining confocal microscopy experiments and numerical simulations. We find that effective interactions between microgels are clearly temperature dependent. In addition, microgel mixtures possess an enhanced stability compared to hard colloid mixtures - a property not predicted by a simple Hertzian model. Based on numerical calculations we propose a multi-Hertzian model, which reproduces the experimental behavior for all studied conditions. Our findings highlight that effective interactions between microgels are much more complex than usually assumed, displaying a crucial dependence on temperature and on the internal core-corona architecture of the particles.

## Abstract (web summary)

Changing temperature of thermoresponsive microgels is typically used as a proxy for modifying volume fraction based on the assumption that doing so does not alter the interaction potential. Bergman et al. shows that this picture is oversimplified and microgels are better described by a multi-Hertzian model.

## Introduction

Microgels are hybrid particles with a dual colloid-polymer nature, belonging to the class of so-called soft colloids^1,2^. A microgel consists of a mesoscopic cross-linked polymer network, which can deform, shrink or interpenetrate with another microgel^3,4^. Often, as a result of the synthesis conditions, a particle possesses a denser core and a more loosely crosslinked corona^5,6^, which also includes so-called dangling ends^7,8^. Microgels are considered smart colloidal materials: in response to external parameters such as temperature, pH, ionic strength, light, or electric field (depending on the nature of the polymers) a particle is able to change its size as well as other connected properties such as the polarizability^9^ or elasticity of the particle^10,11^. Thus, they are promising for and already employed in several applications, such as photonic crystals^12,13^, drug delivery systems^14–16^, or nanotechnologies^17^. In addition, thanks to their high tunability and to their softness, microgels represent ideal model systems to study phase transitions^18–22^ and glass or jamming transitions in dense colloidal dispersions^23–25^.

In the case of thermoresponsive microgels made of poly(N-isopropylacrylamide) (PNIPAM), the soft colloids are swollen below the volume phase transition temperature (VPTT) of 32 °C^26–28^. At temperatures *T* > VPTT, the swollen microgel network collapses and expels a significant fraction of water^27,29^. Thus, temperature is readily used as a convenient parameter to control in situ the size and the volume fraction of microgel samples^18–21,25,30^. In doing so, however, one implicitly assumes that such a temperature change does not alter the effective interactions between microgels. Early studies have proposed to model effective interactions between swollen microgel particles (below the VPTT) in terms of a hard-sphere-like potential, with a modified/effective hard sphere diameter^5,31,32^, whereas for *T* > VPTT microgels should behave as attractive spheres^32^. Recent research shows that the interactions between swollen microgels can be more accurately reproduced by a soft Hertzian repulsion in the fluid region of the phase diagram^33^, while brush-like models can be used for highly packed samples^34,35^. All these different models point to the surprising fact that there is not yet a unifying picture which can describe microgels’ interactions. Clearly there is the need to carefully characterize the interparticle potential under different experimental conditions across the entire phase space. Such a step is necessary not only for a correct use of microgel systems in their widespread applications but also from a fundamental point of view: only fully characterized systems should be used to work on open problems in condensed matter physics, such as glass transition and jamming.

In this work, we investigate the effective interactions of microgels in a wide region of the fluid regime, and as a function of temperature for *T* < VPTT, i.e., for swollen microgels. We study both one-component microgel suspensions and binary mixtures in which much smaller microgels are added, inducing an effective depletion on the large ones. For each state point, experimental structural and dynamical information was compared to its simulated counterpart. We confirm the applicability of a soft repulsive Hertzian interaction potential for the one-component system, even at elevated temperatures. However, the Hertzian model predicts the instantaneous aggregation of the large microgels in the mixtures, which experience a depletion attraction. In contrast, all mixtures are stabilized by the core-repulsion of the microgels. Based on numerical calculations of the effective interaction potential, we develop a multi-Hertzian (MH) model, which ascribes a different elasticity to corona–corona interactions—reflecting the simple Hertzian interactions between microgels at moderate packing fractions—and core–corona or core-core interactions. The MH model captures the structure and the dynamical behavior of the studied binary mixtures at all 48 investigated different state points. Evidently, it is imperative to consider the variation of the interparticle potential upon changing temperature and, crucially, the internal structure of the microgels to correctly describe their behavior, particularly for conditions where microgels are forced together—for example, in electric field applications or in the dense glassy regime, which is most widely studied in the microgel literature. Furthermore, our results raise fundamental questions on the widespread practice to tune the volume fraction via a temperature change without accounting for the different nature of the system.

## Results

### Structure and dynamics of one-component microgel systems

**Fig. 1:**
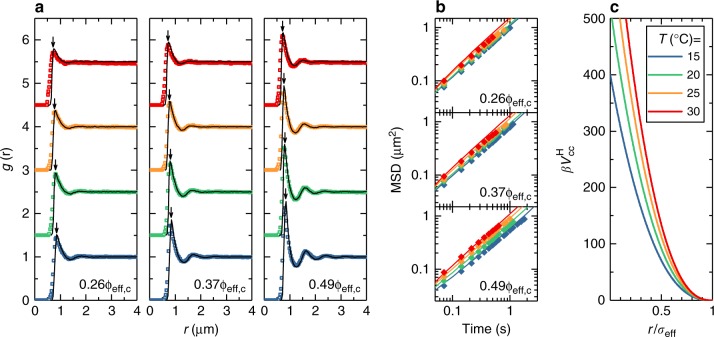
Temperature-dependent structure, dynamics and interaction potential of one-component microgel suspensions. Symbols indicate experimental data, solid lines represent simulations. Color legend applies to all panels. **a** Radial distribution functions *g*(*r*) from simulations and experiments. Panels show data for $\phi _{{\mathrm{eff,c}}}= 0.26,0.37$ and 0.49 (left to right, evaluated at *T*=15°C) for 15°C ≤ *T* ≤ 30 °C. Graphs are offset along the *y*-axis for clarity. Downward pointing arrows indicate the hydrodynamic diameter of colloids at each *T*. **b** MSDs for 15 °C ≤ *T* ≤ 30 °C reconstructed from the *x*,*y* trajectories, i.e. 〈*x*^2^ + *y*^2^〉, with $\phi _{{\mathrm{eff,c}}}=0.26,0.37$ and 0.49 (from top to bottom). **c** Hertzian interaction potential at different temperatures: *U*_cc_ = 400, 520, 640, 760*k*_B_*T* for *T* = 15, 20, 25, 30 °C respectively. The distance is rescaled by *σ*_eff_ = 2*R*_H_

We start by analyzing the behavior of one-component microgel suspensions (also referred to as ‘colloid’ samples) with weight fractions wt% = 2.2, 3.3, 4.4. The radial distribution function (*g*(*r*)) and mean squared displacements (MSD) of the samples were measured at four different temperatures in the range 15–30 °C. We find, as expected, that an increase in particle concentration leads to an increase in the structural correlations for all *T* (Fig. 1a). An increase in temperature is associated with the deswelling of particles, which is quantified by additional dynamic light scattering measurements (Supplementary Fig. 1, Supplementary Note 1), and thus causes  a decrease of the volume fraction of the sample. This leads to a reduced structural order as well as to the shift of the main peak of the *g*(*r*) toward smaller distances. From the trajectories obtained with confocal laser scanning microscopy (CLSM) we also reconstruct the MSDs in a time window within the long-time diffusive regime of the microgels (Fig. 1b). We find that increasing temperature speeds up particle diffusion, due to the reduction in volume fraction as well as to the faster thermal motion and to a reduction in solvent viscosity.

In order to describe the experimental behavior, we use the soft Hertzian-type repulsion which has been previously shown to accurately describe microgel interactions in the fluid phase at 15 °C^33,36^. The (colloid-colloid) Hertzian potential $V_{{\mathrm{cc}}}^{\mathrm{H}}(r) = U_{{\mathrm{cc}}}(1 - r/\sigma _{{\mathrm{eff}}})^{5/2}\theta (\sigma _{{\mathrm{eff}}} - r)$ where *θ*(*r*) is the Heaviside step function, depends on two control parameters: the effective colloid diameter *σ*_eff_ and the interaction strength at full overlap *U*_cc_. We fix the former to be equal to 2*R*_H_, where *R*_H_ is the (experimentally determined) hydrodynamic radius of the particles at each considered *T* (Supplementary Fig. 1, Supplementary Note 1). Next, we adjust the colloid volume fraction *ϕ*_eff,c_ at *T* = 15 °C around the value predicted by viscometry (see Methods, Supplementary Fig. 2, Supplementary Note 2) and we vary *U*_cc_ until a good correspondence is found with the experimental *g*(*r*)s.

We find, in line with previous work with slightly different microgels^33^, that the interaction strength at *T* = 15 °C is *U*_cc_ = 400*k*_B_*T* and the three colloid packing fractions, that will serve as a basis also for the binary mixtures discussed later on, are *ϕ*_eff,c_ = 0.26, 0.37 and 0.49 at 15 °C. To model the variation in temperature, the volume fractions are changed according to the deswelling of the microgels (Supplementary Fig. 1, Supplementary Note 1), and again we vary the interaction strength *U*_cc_ until the experimental radial distribution functions are well reproduced also at higher *T*. Consequently, we find that the numerical and experimental *g*(*r*) are in good agreement for all investigated state points. In particular, the positions of all peaks is well-captured and the secondary peaks are quantitatively reproduced, while some deviations are observed close to the main peak. However, there is no systematic trend of such deviations with respect to packing fraction, as shown and described in Supplementary Fig. 3 and Supplementary Note 3. This suggests that the discrepancy is mostly driven by data noise. A systematic worsening of the agreement is found at *T* = 30 °C, where lower spatial resolution of the CLSM in the *z*-direction and the rapid Brownian motion of the particles leads to a reduction in the peak height and a broadening of the *g*(*r*) data for values to the left of the first peak^33^.

The agreement between experimental and numerical data indicates that the Hertzian model is able to describe the structure of the swollen microgel system in the range of investigated packing fractions. Furthermore, we find that *U*_cc_ approximately follows a linear dependence on temperature (Supplementary Fig. 5, Supplementary Note 5). The fact that raising the temperature makes PNIPAM more hydrophobic might give rise to expectations that by increasing *T* microgels become more and more attractive^29,32^. By contrast, our results show that the Hertzian repulsion is an increasing rather than decreasing function of temperature, at least up to 30 °C (Fig. 1c), in agreement with static light scattering experiments previously obtained with microgels with a lower crosslink density^32^. Only close to the VPT and beyond, outside of the regime explored in this work, attractive interactions become dominant^32^.

A further test of the Hertzian model can be made by comparing experimental and numerical MSDs. To this aim we use Brownian Dynamics (BD) simulations, which show that that the Hertzian model is also able to reproduce the variation of the MSDs (Fig. 1b) with *T* and *ϕ*_eff,c_ in the investigated regime. The direct comparison between the numerical and experimental self-diffusion coefficients is reported in Supplementary Fig. 4 and Supplementary Note 4.

**Fig. 2:**
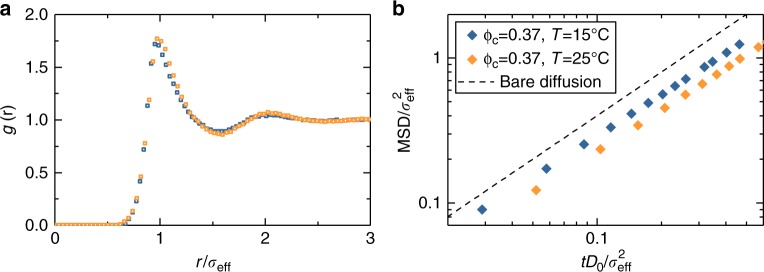
Structure and dynamics for two state points at different temperature with equivalent packing fraction. (**a**) Experimental $g(r)$ and (**b**) MSD for samples with *ϕ*_eff,c_ = 0.37 at two different temperatures (*T* = 15 and 25 °C) corrected for size. *D*_0_ is the zero-colloid limit diffusion coefficient and its associated MSD (dashed line) is also shown

It is particularly interesting to directly compare samples with the same effective volume fraction but at different temperatures, i.e. samples with unequal number density, as shown in Fig. 2. Comparing the experimental data for *g*(*r*), we find a weak but detectable increase of the correlation with increasing temperature. Indeed, at the higher *T* the first two peaks increase their height and shift towards larger separations, the in-between minimum deepens and a weak oscillation beyond *r*/*σ*_eff_ = 2 appears. Not only the structure of the system is affected, but also the dynamics. After appropriately rescaling the data to take into account the different hydrodynamic radii and zero-colloid limit diffusion coefficients at each *T*, the experimental MSDs show a marked difference between the two samples: the *T* = 25 °C system is much slower than the one at lower temperature. Thus, at higher *T* the system is more structured, which is consistent with the stronger Hertzian repulsion that we have determined within our theoretical analysis. However, it is important to stress that the present evidence is based solely on experimental data and does not rely on the particular choice of any model. Our measurements clearly show that the interaction potential between the particles changes as a function of temperature, even within the swollen regime (15–30 °C).

To summarize, we have found that a temperature-dependent Hertzian model can be used to correctly capture both the structure and the dynamics of one-component microgel suspensions in the investigated temperature and packing fraction range. Importantly, the Hertzian repulsion is found to increase with temperature. These findings directly confirm the hypothesis that, by changing the temperature, not only the packing fraction is varied but also the interaction potential is considerably affected. This is particularly important for studies in which the temperature is used as a facile way to tune the effective volume fraction of soft microgels, where these temperature-dependent changes in interparticle interactions should be carefully considered.

### The Hertzian model poorly describes microgel mixtures

We now turn to analyze mixtures of large (colloid) and small (depletant) microgels. The very small size ratio *R*_H,depletant_/*R*_H,colloid_ changes very little, i.e. from 0.055 to 0.060, within the investigated temperature range (Supplementary Fig. 1, Supplementary Note 1). In this framework, it is possible to derive an effective interaction potential for large microgels only, integrating out the small particles’ degrees of freedom^37^. The small microgels thus induce a depletion interaction between the colloids.

**Fig. 3:**
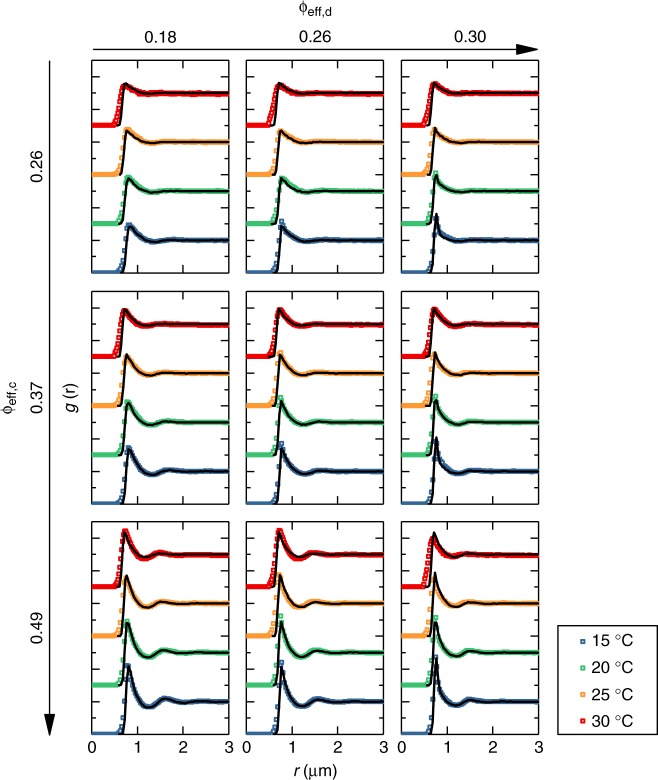
Experimental and numerical structural correlations for all investigated binary mixtures. Experimental *g*(*r*)s (colored squares) are compared to numerical ones (solid lines) based on the multi-Hertzian model. Data for different samples are offset in *y* for clarity. The color legend applies to the entire graph. Values of *ϕ*_eff,c_ and *ϕ*_eff,d_ at 15 °C are given for each row and column, respectively. For higher temperatures, the values of *ϕ*_eff,c_, *ϕ*_eff,d_ can be found in Table 1

We investigate nine colloid-depletant mixtures with colloid wt% = 2.2, 3.3, 4.4 and depletant wt% = 0.26, 0.54, 0.81. We start by analyzing structural correlations (Fig. 3). In the presence of depletants, the colloidal particles show an increased attraction: the first maximum of the *g*(*r*) increases in height and becomes asymmetric. In addition, the nearest neighbor distance, characterized by the position of such maximum, decreases with the addition of depletants.

**Fig. 4:**
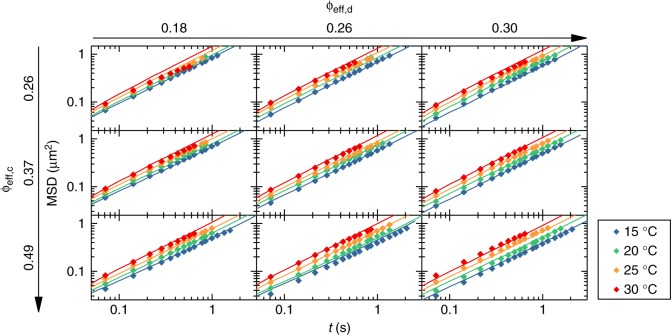
Experimental and numerical mean square displacements for all investigated state points. Diamonds denote 2D experimental data (〈*x*^2^ + *y*^2^〉), while solid lines represent the corresponding simulation results based on the MH model. The color legend applies to the entire graph. Values of *ϕ*_eff,c_ and *ϕ*_eff,d_ at 15 °C are given for each row and column, respectively. For higher temperatures, the values of *ϕ*_eff,c_,*ϕ*_eff,d_ can be found in Table 1

A striking result from the experiments is that the depletion attraction is not as strong as expected: all studied mixtures are surprisingly stable and fluid-like. Comparing with recent results for a binary mixture of hard spheres (colloids) and microgels (depletants), phase separation was observed well within the currently investigated range of depletant concentrations^38^. Furthermore, theoretical models for depletion among soft particles would predict a strongly enhanced depletion attraction as compared to that occurring in corresponding hard-sphere systems^39^. On the contrary, our experimental findings show that the effect of the depletion attraction is small. This is confirmed by the variation of the MSDs with added depletants (Fig. 4): upon increasing depletant concentration we only observe a moderate slowing down of the diffusion.

In order to describe the observed behavior, we start by modeling the binary mixtures at *T* = 15 °C using again the Hertzian model. Thus, the total interaction in the mixture amounts to $V_{{\mathrm{tot}}} = V_{{\mathrm{cc}}}^{\mathrm{H}} + V_{{\mathrm{cd}}}^{\mathrm{H}} + V_{{\mathrm{dd}}}^{\mathrm{H}}$, where the three terms are the direct colloid-colloid interaction, the colloid-depletant interaction and the direct depletant-depletant interaction, respectively. For the first term $V_{{\mathrm{cc}}}^{\mathrm{H}}$, we use the previously established model in the absence of depletants, with interaction strength *U*_cc_ = 400*k*_B_*T*. To estimate the depletant-depletant term we rely on additional static light scattering measurements for the small microgels (Supplementary Fig. 6, Supplementary Note 6), which lead us to an estimated Hertzian interaction strength at contact of $U_{{\mathrm{dd}}} \simeq 100k_{\mathrm{B}}T$.

Assuming additive interactions in the mixture, the cross-interaction strength between large and small microgels would be *U*_cd_ = 250*k*_B_*T*. Since simulations of the full binary system are rather costly at the small investigated size ratios, we proceed by assuming ideal depletant-depletant interactions, which simplifies the theoretical description in terms of an (effective) one-component system. This assumption is justified by the small size as well as by the very soft interactions between depletants. The interactions between large microgels can thus be calculated as $V_{{\mathrm{eff,cc}}}^{\mathrm{H}} = V_{{\mathrm{cc}}}^{\mathrm{H}} + V_{{\mathrm{depl}}}$, where *V*_depl_ is the additional depletion term induced by the small microgels which depend only on the cross-interactions $V_{{\mathrm{cd}}}^{\mathrm{H}}$ and on the depletant volume fraction *ϕ*_eff,d_, as explained in the Methods.

The resulting interaction potential $V_{{\mathrm{eff,cc}}}^{\mathrm{H}}$ is far too attractive (Supplementary Fig. 7a, Supplementary Note 7) even at very low *ϕ*_eff,d_, independently on the choice of *U*_cd_. Indeed, even considering rather low (and strongly non-additive) depletant-colloid interactions, the resulting effective potential would lead to instantaneous aggregation between the colloids. In contrast, all studied binary mixtures are experimentally stable. Thus, the Hertzian repulsion model dramatically fails in capturing the behavior of the particles once we add even the smallest amount of attractive depletion.

### The multi-Hertzian model for microgel-microgel interactions

The Hertzian model fails to describe binary mixtures, because its soft repulsion is too weak to counteract the depletion attraction. Indeed, for soft, penetrable particles, we have to consider interactions down to *r* → 0, where the depletion attraction can become very large^39^. We thus need to model the repulsion between microgels in a more realistic way, taking into account that the density profiles for individual microgels studied in this work show a core-corona structure^5,7,8^. Hence, the addition of depletion interactions allows us to reveal the ‘hidden’ effect of the microgel core even without directly probing too dense regimes.

**Fig. 5:**
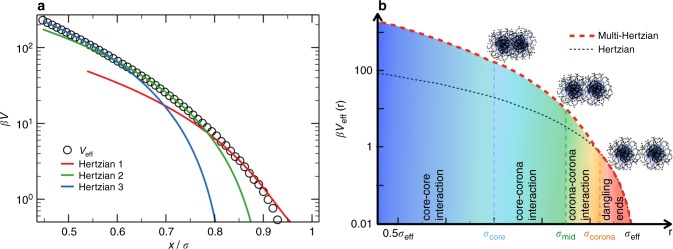
The multi-Hertzian model. **a** Calculated effective potential between two microgels as a function of their center-to-center distance. Lines are fits to three different Hertzian contributions, labeled respectively as Hertzian 1, which corresponds to the calculated elastic moduli^40^ and whose effective diameter σ is used to rescale the *x*-axis, Hertzian 2 and Hertzian 3 representing the contributions of the inner structure of the microgels. For the reported microgel, the fitted strengths are *U*_1_ = 335*k*_B_*T*, *U*_2_ = 1182*k*_B_*T* and *U*_3_ = 2617*k*_B_*T* and the fitted lengths are *σ*_1_ = σ, *σ*_2_ = 0.92*σ* and *σ*_3_ = 0.8354*σ* in good qualitative agreement with the ones used to fit experimental data whose parameters are given in Table 2; **b** the model describing experimental data with the employed interactions lengths: *σ*_core_, below which core-core interactions take place, *σ*_mid_ relevant to the onset of core-corona interactions and *σ*_corona_ which reflect the heterogeneous nature of the outer corona shell. A comparison with the Hertzian model is also provided. Note the logarithmic scales on the *y*-axis for both panels

In a recent numerical work, some of us have addressed the question of the validity of the Hertzian model by performing numerical simulations of realistic in silico microgel particles^8,40^. We have shown that the Hertzian predictions only hold up to repulsion strengths of ≈ 6*k*_B_*T* and to packing fractions of order unity. These results confirm that, for one-component microgels in the range of *ϕ* investigated here, we can successfully describe the system properties with the Hertzian model, with the strength of the repulsion being linked to the elastic moduli of the microgels, which can be computed independently^40^. For smaller separations, when the repulsion between two microgels sensibly exceeds the thermal energy, the interaction acquires a clear non-Hertzian nature, as shown in Fig. 5a. Interestingly, here we find that the full dependence of the effective interaction on the microgel-microgel separation can be fitted to a cascade (three in the example shown in Fig. 5) of Hertzian potentials. The very good quality of the fit can be understood in terms of the microscopic architecture of the microgel, which can be considered to be composed by a sequence of more and more dense shells, each of them corresponding to a different internal elasticity and thus to a different Hertzian contribution. Thus, while the Hertzian model is only able to capture the interactions between the outer parts of the coronas of the two microgels, stronger repulsions need to be considered in order to include core-corona and core-core contributions. Such a multi-Hertzian model is able to describe the numerically calculated potential up to the smallest simulated particle-particle distances, which correspond to strengths of order 200*k*_B_*T* for the considered microgel.

We thus apply the MH model (schematically illustrated in Fig. 5) to the investigated binary mixtures, fixing most of the model parameters according to experimental data as described in Methods. It turns out that we need to take into account four successive shells, reading as

1 $${\begin{array}{*{20}{l}} {V_{{\mathrm{cc}}}^{{\mathrm{MH}}}(r)} \hfill & = \hfill & {U_{{\mathrm{cc}}}(1 - r/\sigma _{{\mathrm{eff}}})^{5/2}\theta (\sigma _{{\mathrm{eff}}} - r) + U_{{\mathrm{corona}}}(1 - r/\sigma _{{\mathrm{corona}}})^{5/2}\theta (\sigma _{{\mathrm{corona}}} - r)} \hfill \\ {} \hfill & {} \hfill & { + U_{{\mathrm{mid}}}(1 - r/\sigma _{{\mathrm{mid}}})^{5/2}\theta (\sigma _{{\mathrm{mid}}} - r) + U_{{\mathrm{core}}}(1 - r/\sigma _{{\mathrm{core}}})^{5/2}\theta (\sigma _{{\mathrm{core}}} - r)} \hfill \end{array}}$$

where the outermost shell, extending up to *σ*_eff_, coincides with the Hertzian model of strength *U*_cc_. This ensures that, in the investigated regime, the behavior of the one-component microgel system is the same for the MH model and for the Hertzian model (Supplementary Fig. 8, Supplementary Note 8). The size of the innermost shell is set by *σ*_core_, which is the experimentally determined core size and indicates the onset of core-core interactions with a very large repulsion strength *U*_core_. Because the transition from the core to the corona is gradual, we introduce an intermediate shell at the midpoint *σ*_mid_, signaling core-corona interactions. We find that the introduction of an additional elasticity within the outer shell (starting at *σ*_corona_, the midpoint of the corona) is necessary to reproduce the experimental data to differentiate the contribution of the dangling ends^7^ of the order of $\sim k_{\mathrm{B}}T$ from the corona one. This turns out to be slightly different from the numerical result in Fig. 5a, probably due to the small size of the investigated microgels in simulations and to the absence of true dangling ends in this representation. At each of the characteristic lengths of the potential (see Fig. 5b), an associated interaction strength is estimated by simple arguments (see Methods), except for *U*_corona_, which is adjusted to match the experimental data. The obtained strengths are in qualitative agreement with those resulting from the MH fit of the calculated effective potential.

### Developing the multi-Hertzian model at 15 °C

We apply the MH model to binary mixtures of large (‘colloid’) and small (‘depletant’) microgels. The resulting effective potential is now the sum of the multi-Hertzian model for the direct colloid-colloid interactions and the depletion term, as $V_{{\mathrm{eff,cc}}}^{{\mathrm{MH}}} = V_{{\mathrm{cc}}}^{{\mathrm{MH}}} + V_{{\mathrm{depl}}}$. The latter term contains the cross-interactions between the two types of microgels (colloid-depletant interactions), which for consistency should also take a multi-Hertzian form. However, we have explicitly checked that its inclusion makes no significant difference to using a simple Hertzian. Rather, it complicates the description, so that we stick to the simple Hertzian $V_{{\mathrm{cd}}}^{\mathrm{H}}$, whose strength *U*_cd_ has yet to be determined. Also, we need to determine the effective depletant volume fraction *ϕ*_eff,d_.

We start by considering *T* = 15 °C and simultaneously vary the free parameters *U*_corona_, *U*_cd_ and *ϕ*_eff,d_ until we find an optimal agreement to reproduce the measured *g*(*r*). The resulting effective potential which best describes the experimental data is found for *U*_corona_ = 8.25 × *U*_cc_ = 3300*k*_B_*T* and *U*_cd_ = 80*k*_B_*T* (Supplementary Fig. 7b, Supplementary Note 7). Effective depletant volume fractions are *ϕ*_eff,d_ = 0.18, 0.26, 0.30 at 15 °C. The calculated *g*(*r*)s are shown as lines in Fig. 3 and are found to reproduce the behavior of the measured data for all depletant and colloid volume fractions at the examined temperature. Particularly noteworthy is the development of an asymmetric main peak of *g*(*r*) at the highest studied volume fractions, which is accurately captured by the MH model.

These findings point out the strongly non-additive character of the interactions in microgel mixtures: indeed the colloid-depletant *U*_cd_ is significantly lower than the average of the two individual interactions for colloid and depletant microgels. This is probably due to the ability of soft particles to deform or overlap with each other, differently from hard particles. A possible explanation is that the very small depletant here involved can quite freely interpenetrate within the corona of the large ones, modifying the cross-interactions. The non-additivity is thus the key ingredient which allows us to explain the surprising stability of our soft binary mixtures^41,42^. Indeed, thanks to this feature, particles are able to experience a much more moderate depletion attraction than what is observed in hard colloid-soft depletant mixtures^38^ and in additive soft ones^39^. Hence our soft mixtures will eventually phase separate only at much larger depletant concentrations.

### Using the multi-Hertzian model at higher temperatures

The incorporation of the temperature dependence is a first real test to the robustness of the MH model. With increasing temperature, the interactions between the colloids in the binary mixtures change. Increasing the temperature has an effect not only on all interactions in the MH model, but also on the colloid-depletant cross interaction *U*_cd_ and on the effective volume fractions *ϕ*_eff,c_ and *ϕ*_eff,d_. The two-volume fractions are easily dealt with: the deswelling of the microgels (in both cases) automatically yields the volume fractions at higher temperatures (see Table 1). For the MH model parameter estimate, we use the temperature dependence of the Hertzian term (see also Fig. 1b) for the outermost corona. We further note that the core size is temperature independent, based on previously published experimental data^33^ and our own unpublished work. Thus, the intermediate shells in the MH model become thinner and their associated strengths are chosen as done for 15 °C. A detailed description of the choice of parameters is given in the Methods section, but it is important to stress that the temperature dependence of the MH model has zero free parameters: everything is fixed based on experimental data and the parameters found for 15 °C. Thus the only parameter left to vary is the colloid-depletant cross interaction *U*_cd_. Once a good agreement with experimental data is found, it is checked a posteriori that the estimated values are very reasonable and obey a roughly linear relation to temperature, analogously to *U*_cc_ (Supplementary Fig. 5, Supplementary Note 5).

**Table 1:** Summary of effective volume fractions for all samples at all temperatures

*T* (°C)	one-component system	binary mixtures		
	*ϕ*_eff,c_	*ϕ*_eff,d_	*ϕ*_eff,d_	*ϕ*_eff,d_
15	0.26	0.18	0.26	0.30
15	0.37	0.18	0.26	0.30
15	0.49	0.18	0.26	0.30
20	0.22	0.17	0.24	0.28
20	0.315	0.17	0.24	0.28
20	0.42	0.17	0.24	0.28
25	0.19	0.16	0.23	0.26
25	0.275	0.16	0.23	0.26
25	0.37	0.16	0.23	0.26
30	0.155	0.145	0.215	0.24
30	0.22	0.145	0.215	0.24
30	0.29	0.145	0.215	0.24

The experimental *g*(*r*)s for the binary mixtures are compared with the simulated data in Fig. 3 for all investigated *T*. The final model parameters are reported in Table 2. We find that the MH model captures all the distinct features of the depletion attraction: the peak shift, its increase and asymmetry all emerge with increasing *ϕ*_eff,d_ (Fig. 3). It is worth to stress that the agreement of the model with experiments spans 48 different state points and is based essentially on adjusting two parameters: the strength of the second corona shell *U*_c*orona*_ (only determined at 15 °C) for the MH model and the cross-interaction strength *U*_cd_ (adjusted at each temperature) for the depletion interaction. Thus the present findings represent a strong test in favor of the validity of the present model.

**Table 2:** Summary of all parameters for the MH model and depletion term as a function of temperature

*T* (°C)	*U*_core_	*U*_mid_	*U*_corona_	*U*_cc_	*U*_cd_	*σ*_core_	*σ*_mid_	*σ*_corona_	*σ*_eff_
15	10 000	4 000	3 300	400	80	0.70	0.85	0.925	1
20	10 000	5 200	4 300	520	108	0.74	0.87	0.935	1
25	10 000	6 400	5 300	640	144	0.77	0.885	0.945	1
30	10 000	7 600	6 300	760	180	0.83	0.915	0.960	1

Hertzian strengths *U*_core_, *U*_mid_, *U*_corona_ and *U*_cc_ in *k*_B_*T* and lengths *σ*_core_, *σ*_mid_,*σ*_corona_ and *σ*_eff_ (in units of *σ*_eff  _) as used for colloid-colloid interactions. Hertzian strength *U*_cd_ in *k*_B_*T* used to describe the colloid-depletant (non-additive) interaction in binary mixtures

**Fig. 6:**
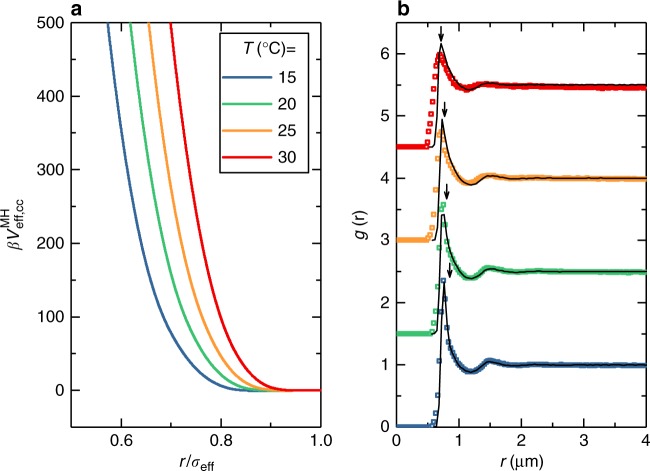
Typical $V_{{\mathrm{eff,cc}}}^{{\mathrm{MH}}}$ and resultant *g*(*r*)s for one state point at different temperatures. **a** Calculated effective potential $\beta V_{{\mathrm{eff,cc}}}^{{\mathrm{MH}}}$ based on temperature-dependent $V_{cc}^{{\mathrm{MH}}}$ and *V*_cd_. At *T* = 15 °C, $\beta V_{{\mathrm{eff,cc}}}^{{\mathrm{MH}}}$ displays a shallow negative minimum at $\sim - 0.6k_{\mathrm{B}}T$. (**b**) Comparison between numerical *g*(*r*)s (solid lines) and experimental *g*(*r*)s (colored squares). From top to bottom temperature increases from 15 to 30 °C. Downward pointing arrows indicate the hydrodynamic diameter of colloids which does not generally coincide with the first peak position at any temperature

In order to better visualize the effect of temperature, Fig. 6 shows the results for the state point with the largest colloid and depletant volume fractions (*ϕ*_eff,c_ = 0.49 and *ϕ*_eff,d_ = 0.30 at 15 °C). An increase of *T* again reduces the structural correlations and also the effect of depletion (due to the smaller effective depletant volume fraction), which manifests itself at each temperature by an increased asymmetry and by a shift of the main peak of the *g*(*r*) toward smaller values of *r* compared to the one-component system. The peak position is found at smaller distances with respect to the hydrodynamic radius of the colloids, clearly indicating that the particles partially overlap. The agreement of the MH model with experiments becomes worse for 30 °C, similarly to the case of the one-component system and probably due to the larger statistical noise in the experimental values. The average deviations between numerical and experimental curves are reported in Supplementary Fig. 3 and Supplementary Note 3.

A second robustness test for the MH model is carried out by calculating the MSD and comparing it with experiments. Similarly to what has been done for the one-component systems, we rely on BD simulations and compare the calculated and measured MSD of large microgels for each of the nine mixtures in the temperature range 15C ≤ *T* ≤ 30 °C. As shown in Fig. 4, the current model is also able to capture the particle dynamics for all studied state points. This is confirmed in Supplementary Fig. 4 and Supplementary Note 4, where the self-diffusion coefficients for all state points are shown and described. The small deviations between experiments and simulations observed at 30 °C can again be rationalized by the larger tracking errors associated to the rapid Brownian motion of the microgels at this temperature.

## Discussion

In this study, we have presented an extended investigation of microgel suspensions in a three-axis phase diagram. In addition to varying microgel volume fraction and temperature, we also varied the concentration of a second component in the suspension, namely smaller microgels, which act as depletants. We investigate one-component and binary mixtures of microgels in a wide range of control parameters, amounting to 48 different state points. Through the combination of confocal microscopy experiments and simulations, we provide a systematic and comprehensive characterization of both static and dynamic observables in the form of radial distribution functions and mean-squared displacements of the large microgels. Based on explicit calculations of the effective potential between two microgels, we have been able to develop a new interaction potential that, with a single set of experiment-informed parameters, is able to reproduce the statics and dynamics of real microgel suspensions, accounting for the dependence on microgel volume fraction, temperature and depletant concentration. Although microgels are nowadays a widely studied model system, such an extensive study was crucially missing. The several novel findings reported here will change the approach to the use of microgels as model systems in future work. Indeed, these soft particles appear to be much more complex systems than naively thought.

First of all, we have provided evidence that the effect of temperature on microgel-microgel effective interactions is not negligible, even within the swollen regime only. The soft Hertzian repulsion between the particles becomes steeper with increasing *T*. This seemingly straightforward result is not obvious since, for *T* > VPTT, microgels become attractive due to the increased van der Waals and additional hydrophobic interactions. Therefore, an increase of repulsion goes in the opposite direction. The trend can be rationalized by thinking of the microgels only in physical terms (ignoring polymer-solvent interactions which are not yet dominant): as the particles become smaller, they also become more compact and hence somewhat less penetrable. A further change in interactions at high *T* is however hinted by the present results, as the simple repulsive model that we have adopted shows increasing deviations at the highest studied *T* = 30 °C, approaching the VPTT at 32 °C. Close to the VPTT, a much more careful evaluation, also in terms of charge effects which could become important as shown by our and others preliminary measurements^43^, will be required. For the examined *T*-interval, the present findings clearly show that the variation of volume fraction that is obtained by changing *T*, a commonly used method in experiments to efficiently explore a larger portion of the phase diagram, should be done with caution, as doing so significantly affects the effective interactions between the particles. Previous works have already pointed out this important aspect through indirect observations^29^, but here for the first time we provide a direct evidence and quantify the change of behavior with *T* across the swollen regime.

Secondly, we have shown that a simple structureless model such as the Hertzian repulsion does not work to describe conditions where overlaps between particles and/or deformations start to be probed. These effects are an important physical ingredient that deeply affect the behavior of soft colloids in general and of microgels in particular, at the heart of a large research activity on glass transition and jamming of soft particles. Even without directly exploring dense conditions, the use of depletants has allowed us to probe the effective interactions between microgels at short separation distances, finding evidence of the importance of the internal microgel architecture. We have thus transferred our previous knowledge from a simple Hertzian model to a multi-Hertzian one, which is confirmed by explicit calculations of the effective potential between two microgels, that involves the inclusion of inner shells of different elasticity. Interestingly, we find that to successfully describe the experimental data it is important not only to differentiate between core and corona, but also to take into account the heterogenous character of the corona, further differentiating the contribution of the dangling ends^7,8^. Given the numerous studies where different synthesis protocols have been implemented to obtain other internal structures and crosslink density distributions, see *e.g*. refs ^44–47^, it will be interesting to systematically study and quantify how the crosslink density and internal structure of the microgels influence their effective interactions in future studies.

The multi-Hertzian model that we have designed is based on numerical evidence and on available experimental parameters. The comparison with experimental data has allowed us to determine the unknown parameters, most importantly the cross-interactions between small and large microgels. This is a key player in the depletion interaction, and the very low strength that we have determined for cross interactions does explain the striking finding that soft microgel mixtures are much more stable, up to very high depletant concentrations, than expected. Indeed, previous works with additive soft mixtures have shown how softness enhances depletion attraction^39^. Here we show that this does not happen, because softness allows deformation and interpenetration, which translates to strongly non-additive interactions. It will be interesting to confirm these findings also for other soft mixtures and in particular, for the more studied classical case of soft colloids and non-adsorbing polymers acting as depletants.

Finally, our phenomenological approach will have to be generalized to deal with different conditions such as even higher *T* or larger microgel volume fractions, approaching the glass transition. However, the reported evidence clearly shows that future studies will have to explicitly take into account temperature dependence and internal microgel structure to meaningfully describe microgel behavior and to use them as model systems for exploring phase transitions and glassy dynamics.

## Methods

### Synthesis

PNIPAM particles were synthesized via precipitation polymerization^27,28^. NIPAM was re-crystallized in hexane and all other chemicals were used as received. For the large fluorescent microgels (referred to as colloids), 2.004 g N-isopropylacrylamide (NIPAM, Acros Organics) was dissolved in 82.83 g of water. 0.136 g (4.98 mol% with respect to NIPAM) of the cross-linker N,N-methylenebis(acrylamide) (BIS, Sigma-Aldrich) was added. 0.002 g methacryloxyethyl thiocarbonyl Rhodamine B dissolved in 10 g of water was added to the reaction mixture to covalently incorporate fluorescent sites. The reaction mixture was heated to 80 °C and bubbled with nitrogen for 30 min. The reaction was then kept under a nitrogen atmosphere. To start the reaction, 0.1 g KPS in 5 g water was injected to the mixture. The reaction was then left for 4 h before the heat was turned off and the solution was left to cool down under constant stirring.

For the small non-fluorescent particle synthesis (referred to as depletants), we followed the same procedure. We combined 1.471 g of NIPAM, 0.0647 g (3.2 mol% with respect to NIPAM) of BIS in 96.29 g water. 0.1929 g of sodium dodecyl sulphate (SDS, Duchefa Biochemie) was also added to induce the formation of particles with smaller radii. The mixture was heated to 70 °C, bubbled with nitrogen and 0.0539 g of KPS in 2.0145 g of water was added to start the reaction. The reaction was then left for 6 h under a nitrogen atmosphere.

The particle suspensions were cleaned by three centrifugation and re-dispersion series before the suspensions were freeze-dried to remove all water.

### Sample preparation

All samples were prepared using the freeze-dried microgels and deionised water (purified with a MilliQ system), as this allows us to control the weight concentration. In order to ensure homogeneous dispersions, samples were thoroughly mixed by vortexing and sonication followed by placing the dispersion on a tumbler for two weeks prior to any experiment.

Using this approach, samples with a wt%-range from 0.1 to 1 wt% of colloids and samples with wt%-range from 0.1 to 0.8 wt% of depletants were prepared for viscometry experiments. Very dilute colloid and very dilute depletant suspensions (<0.1 wt%) were made for DLS characterization. Suspensions were diluted until almost completely transparent to avoid multiple scattering. For the SLS measurements, samples with a wt%-range from 0.05 to 0.65 wt% depletant were prepared. For the CLSM experiments, we aimed for binary mixtures with effective colloid volume fraction *ϕ*_eff,c_ = 0.2, 0.3, 0.4, and with additional effective depletant volume fraction *ϕ*_eff,d_ = 0, 0.1, 0.2, 0.3 at 15 °C. As an initial guess for the packing fraction of the samples, we used the shift factor *k* = *ϕ*/wt% as determined from viscometry measurements on colloid-only and depletant-only samples (see below for the experimental *k*-values). The binary mixtures contained colloid wt% 2.2, 3.3 and 4.4 and depletant wt% 0, 0.26, 0.54, 0.81. Final *ϕ*_eff_ were determined by fitting *g*(*r*) curves, as discussed in the manuscript.

### Experiments

The viscosity of colloid-only and depletant-only samples with known wt%-concentration was recorded using an Ubbelohde viscometer at 15 and 30 °C. Flow times were measured 5–6 times, averaged and divided by the flow time of a water sample to extract the relative viscosity of the samples. The relative viscosity was fitted to the well-known Batchelor equation which holds for colloids in the dilute regime^48^: $\eta _{{\mathrm{rel}}} = 1 + 2.5\phi _{{\mathrm{eff}}} + 5.9\phi _{{\mathrm{eff}}}^2$ with *ϕ*_eff_ = *k* × wt%. From these fits the shift factor *k* was determined for 15 and 30 °C. For 20, 25 °C, the data was interpolated. *k*_colloid_ = 0.091, 0.0758, 0.061, 0.046 wt%^−1^, *k*_depletant_ = 0.332, 0.292, 0.253, 0.214 wt%^−1^ for 15–30 °C respectively. The shift factor was used in sample preparation to estimate *ϕ*_eff_.

Microgels were characterized using dynamic light scattering (DLS) with a goniometer-based light scattering instrument that employs pseudo 2D-cross correlation (3D DLS Spectrometer, LS instruments, Switzerland) with laser wavelength *λ* = 660 nm. DLS measurements were performed over a range of 15–30 °C resulting in a swelling curve for both colloids and depletants. The hydrodynamic radii were extracted using a first order cumulant analysis averaged over an angular range of 60–100°, and measured every 10°. To probe the interactions between depletants, static light scattering experiments were performed at several packing fractions, and the small wavevector limit *S*(0) of the static structure factor *S*(*q*) for the small microgels was also obtained.

The binary mixtures were imaged in a Leica SP5 confocal microscope at a frame rate of 13.9 Hz in the range of 15–30 °C. An excitation wavelength of 543 nm was used in combination with an oil immersion objective at ×100 magnification and numerical aperture 1.4. The confocal microscope is housed in a temperature regulated box which provides a temperature control with a stability of ± 0.2 °C over the range of temperatures used. Because scanning in the *z*-direction would have been too slow, we made *xyt*-videos. Such videos of 512 × 512 × 4000 frames were obtained for at least five different positions in the sample to minimize the effects of local density fluctuations. Videos were taken at $\gtrsim 5$ particle diameters away from the glass to avoid wall influences. The accuracy of the coordinates is estimated to be Δ*x* ≈ Δ*y* ≈ 11 nm^33^. Using standardized image analysis and particle tracking routines^49^, the 2D *g*(*r*)s and 2D mean square displacements (MSDs) (〈*x*^2^ + *y*^2^〉) were obtained. To ensure the 2D *g*(*r*) corresponds to the 3D *g*(*r*), the approach as described in Mohanty et al. was employed^33^. In brief, a thinner ‘slice’ of data is created by rejecting out of focus particles, i.e. we only take particles with *z* = 0. Even so, there will always remain some variation in the *z*-position of the tracked particles. This has been taken into account in the numerical calculations by adding a suitable noise along one of the axes.

### Model and theory

We consider two systems: one-component microgel systems and binary mixtures. Colloids experience a direct colloid-colloid interaction that we model as Hertzian or multi-Hertzian (MH) as described in the manuscript. The presence of depletants leads to an additional attractive interaction between the colloids. Thus, the effective colloid-colloid interaction potential *V*_eff,cc_ is the sum of two contributions: *V*_cc_ (direct colloid-colloid interaction) and *V*_depl_ (depletion interaction), i.e. *V*_eff,cc_(*r*) = *V*_cc_(*r*) + *V*_depl_(*r*). We assume that depletants are ideal. Under this assumption, the Fourier components of the additional depletion term can be calculated for any colloid-depletant interaction *V*_cd_ as in ref. ^50^

2 $$- \beta \tilde V_{{\mathrm{depl}}}(k) = \rho _d\left[ {{\int} {\mathrm{d}}{\mathbf{r}}\left( {e^{ - \beta V_{{\mathrm{cd}}}({\mathrm{r}})} - 1} \right)e^{{\mathrm{i}}{\mathbf{k}} \cdot {\mathbf{r}}}} \right]^2$$

Here *ρ*_d_ is the reservoir depletant number density and *β* = 1/(*k*_B_*T*). In our study, we consider *V*_cd_(*r*) to be a Hertzian potential. After Fourier transforming Eq. (2) we obtain *βV*_depl_(*r*) which is added to *βV*_cc_ at each considered temperature for the binary mixtures to obtain the total interaction potential *βV*_eff,cc_. We further check that the use of a multi-Hertzian model for *V*_cd_(*r*) does not yield a noticeable change on the obtained results.

The MH model is built up as follows. The outer shell corresponds to the Hertzian soft repulsion: *U*_cc_ = 400, 520, 640, 760 *k*_B_*T* for 15, 20, 25, 30 °C which sets in at *r* = *σ*_eff_, where *σ*_eff_ = 2*R*_H_ is of course temperature dependent (see also Supplementary Fig. 1 and Supplementary Note 1). The inner shell corresponds to the core and is temperature independent. We estimate the core diameter as *σ*_core_ = 0.7*σ*_eff_ thanks to available experimental SAXS data (and related fuzzy-sphere model fits) for similar microgels^33^. We fix *U*_core_ = 10^4^*k*_B_*T*, a value compatible with elasticity arguments^1^ which takes into account the high crosslink density in the core. In this way, the innermost and outermost Hertzian terms are completely specified based on experimental data. Since the border between the dense core and loosely crosslinked corona is not so well-defined, we introduce an intermediate point at the midpoint between these two lengths, i.e. *σ*_mid_ = 0.5(*σ*_core_ + *σ*_eff_) = 0.85*σ*_eff_. The associated repulsion strength is arbitrarily chosen to be *U*_mid_ = 10 × *U*_cc_. We find that it is crucial to describe the corona by using two distinct shells, introducing a second intermediate point *σ*_corona_ = 0.5(*σ*_mid_ + *σ*_eff_) = 0.925*σ*_eff_ with its associated strength *U*_corona_. The latter is determined at 15 °C to be 8.25 × *U*_cc_ by comparing simulation results with the experimental *g*(*r*)s. This relation and that for *U*_mid_ = 10 × *U*_cc_ are then kept throughout the temperature range.

The effective potential *V*_eff,cc_ is then calculated at several depletant volume fractions starting with our initial guess from viscometry. Because of the uncertainty in the experimental packing fraction, we adjust *ϕ*_eff,d_ in the simulations at 15 °C until we find good agreement with the experimental data. As described in the manuscript, *ϕ*_eff,c_ was adjusted in the Hertzian simulations at 15 °C. The thus obtained parameter values for the volume fractions (*ϕ*_eff,c_, *ϕ*_eff,d_) and the parameters for the MH model are summarized in Tables 1 and 2, respectively.

To justify the assumption of ideal behavior of small microgels and the non-additive character of interactions in the mixture, we have further quantified the interactions between small microgels assuming a Hertzian repulsion. To calibrate its strength, we have computed *S*(0) by solving the Ornstein-Zernike equation within the Rogers-Young closure, finding that a very soft interaction between depletants, i.e. $U_{{\mathrm{dd}}} \simeq 100k_{\mathrm{B}}T$, captures the small microgels behavior. This estimate is consistent with scaling arguments of the Hertzian model as a function of particle size^51,52^ with respect to the large microgels.

### Numerical calculation of the effective potential

Microgel configurations are built as disordered, fully-bonded networks generated as in ref. ^8,40^ using ≈5000 monomers of diameter *σ*_m_ in a spherical confinement of radius *Z* = 25 *σ*_m_ and crosslinker concentration *c* = 5%. Monomers are in the swollen regime and interact through the classical bead-spring model for polymers^53^.

To calculate the microgel-microgel effective interactions we combine two methods, as described in ref. ^40^. We perform umbrella sampling simulations in which we add an harmonic biasing potential acting on the centres of mass of the two microgels^54^ for small separation distances *r*. The resulting effective potential is calculated as *V*(*r*) = −*k*_B_*T*ln[*g*(*r*)]. At larger values of *r*, we employ a generalized Widom insertion scheme^55^ which is very efficient in sampling the small-deformation regime.

### Bulk simulations

We perform Langevin dynamics simulations of *N* = 2000 colloid particles of mass *m* interacting with the generated effective interaction potential *V*_eff,cc_. The units of length, energy, and mass are *σ*_eff_, *k*_B_*T* and *m* respectively. Time is measured in units of $\sqrt {m\sigma _{{\mathrm{eff}}}^2/k_{\mathrm{B}}T}$. The integration time-step is fixed to 0.001. With this scheme, particles after an initial microscopic time follow a Brownian Dynamics (BD) due to the interactions with a fictitious solvent^56^. The solvent effective viscosity enters in the definition of the zero-colloid limit self-diffusion coefficient *D*_0_, which is the key parameter in BD simulations.

Since the viscosity of the real samples changes for each *T* and for each *ϕ*_eff,d_, we have estimated the experimental *D*_0_ at *ϕ*_eff,d_ = 0 by means of Stokes-Einstein relations using the measured hydrodynamic radii at each *T*. Furthermore, we have also estimated *D*_0_ in the presence of depletant thanks to the viscosity measurements described above. Ideally, one would need to directly use these values in the simulations, but this leads to an incorrect description of the system at low enough colloid packing fractions because BD simulations do not include hydrodynamic interactions, whose effects are strongest in this regime. Hence we have adopted the following strategy: at fixed *ϕ*_eff,c_ and for each *T* and *ϕ*_eff,d_ (i.e., for 16 of our samples), we performed several BD runs in order to select the values of *D*_0_ providing a good agreement for the long-time MSD with experiments. We thus find a unique shift factor on the time axis for all simulated state points, needed in order to convert simulation time into experimental time. Then, the estimated *D*_0_ values were kept fixed for all studied *ϕ*_eff,c_, i.e., for the remaining 32 studied samples no further adjustment was made. The estimated *D*_0_ can thus be considered the effective bare self-diffusion coefficients of our approximated BD approach, and was finally compared to the experimental estimates, finding a good agreement at large depletant concentrations and low temperatures (as reported in Supplementary Fig. 9 and Supplementary Note 9), that is, for the state points where hydrodynamic interactions are less important.

Simulations were performed with particles possessing a polydispersity of 4% with a Gaussian distribution, similar to the experimental system. Slices through configurations of 100 independent state points were used to calculate the radial distribution function *g*(*r*) of the 3D data with sufficient statistics. The *z*-position of particles is randomly displaced by Gaussian noise with a standard deviation of 0.005. In this way, *g*(*r*)s can be successfully compared to the 2D-*g*(*r*) obtained from experiments, as demonstrated in ref. ^33^.

### Code availability

The computer codes used for the current study are available from the corresponding authors on reasonable request.

## Electronic supplementary material

Supplementary Information

## Data Availability

The authors declare that all data supporting the findings of this study are available within the article and its Supplementary Information files. All other relevant data supporting the findings of this study are available from the corresponding authors on request.
