# Supplementary material for: A new look at effective interactions between microgel particles
Source: Nat Commun. 2018 Nov 28;9:5039. doi: 10.1038/s41467-018-07332-5 (PMC6262015; doi:10.1038/s41467-018-07332-5)
Supplement: Supplementary file 1 — Supplementary Information [file 41467_2018_7332_MOESM1_ESM.pdf]

Supplementary Information for: A new look at effective interactions between microgel particles

Bergman et al.

Supplementary Figures

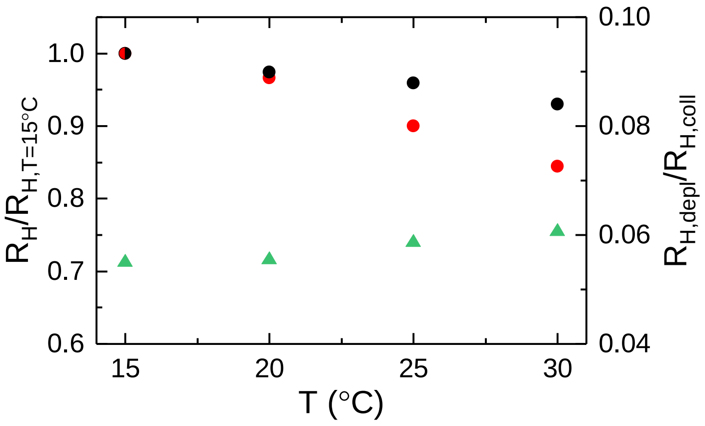

Supplementary Figure 1: Swelling curves and size ratio. Left axis: Relative swelling behaviour for colloid particles (red circles) for which $R_{\mathrm{H},T=15^{\circ}\mathrm{C}} = 425$nm and depletant particles (black circles) for which $R_{\mathrm{H},T=15^{\circ}\mathrm{C}} =$ 23nm. Right axis: size ratio $R_{\mathrm{H,depletant}}/R_{H,\mathrm{colloid}}$ (green triangles) as function of temperature.

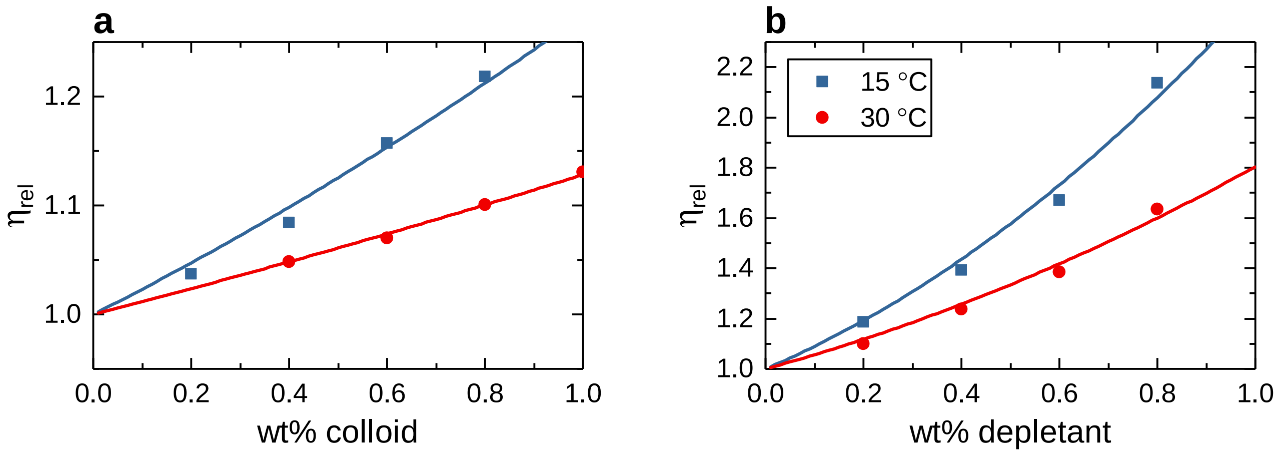

Supplementary Figure 2: Experimental viscometry data acquired for a) colloid only samples and b) depletant only samples. The relative viscosity was measured at 15 and 30°C (blue and red symbols) and fitted with the Batchelor equation (solid lines).

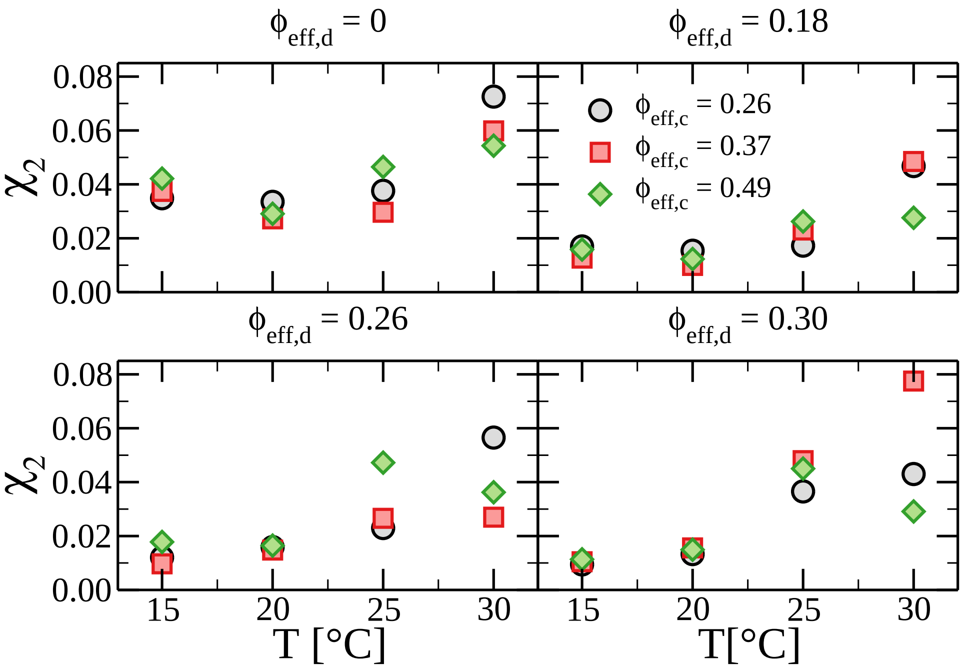

Supplementary Figure 3: The average deviation χ^2 between $g_{\rm sim}$ and $g_{\rm exp}$ for all the investigated state points.

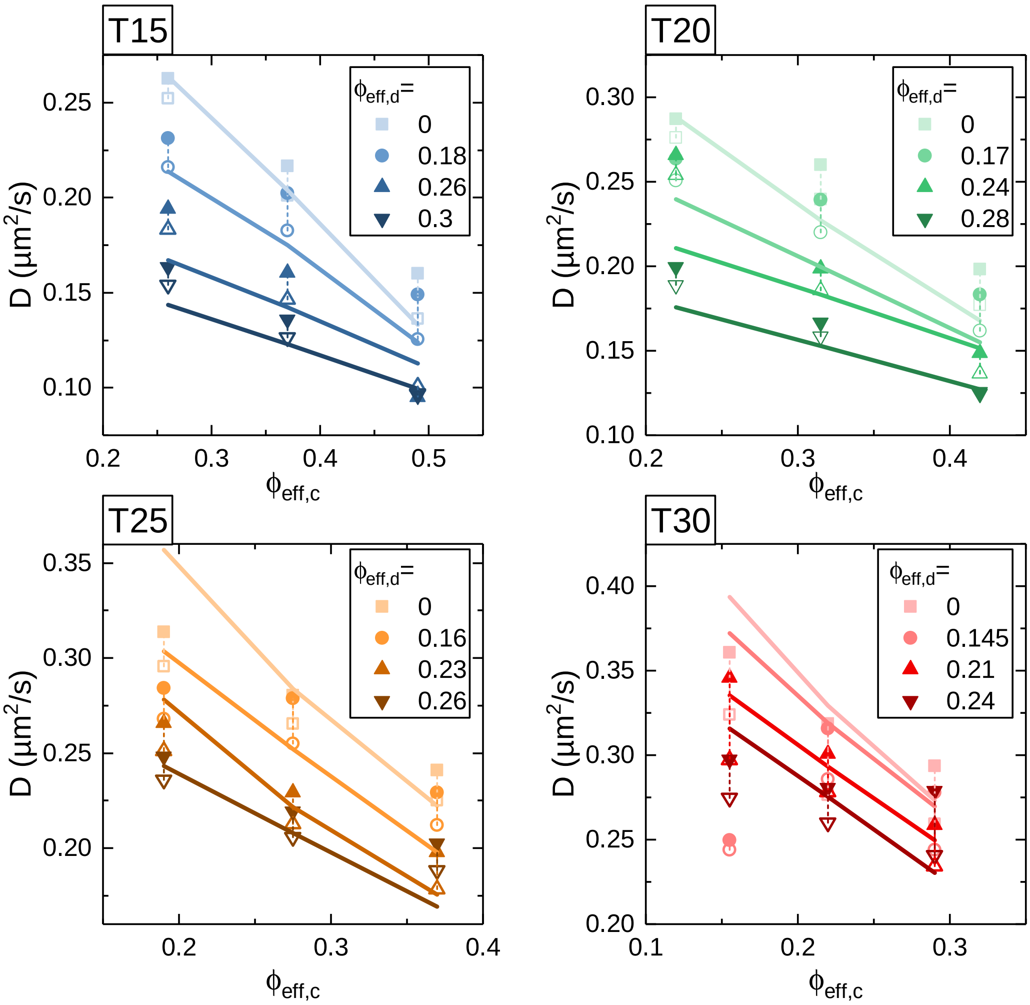

Supplementary Figure 4: Self diffusion coefficients D for each investigated state point plotted versus $\phi_{\mathbf{eff,c}}$. Symbols denote experimental data, lines represent simulated data. The diffusion coefficient taken from the slope of the MSD is shown as open symbols. The diffusion coefficient taken from the Van Hove self-correlations is shown as filled symbols.

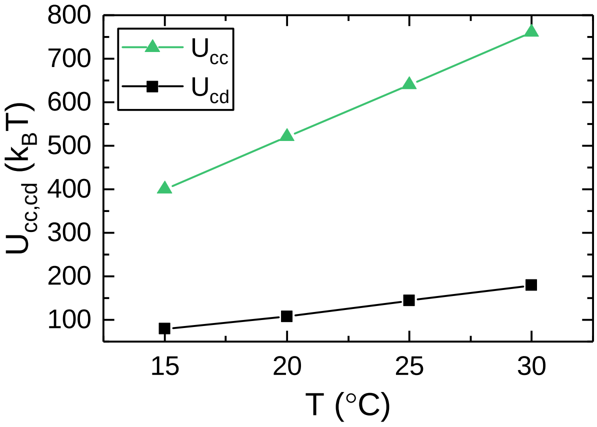

Supplementary Figure 5: Dependence of interactions strength on temperature. Both U_{cc} (green triangles) and U_{cd} over temperature (black squares) are roughly linear.

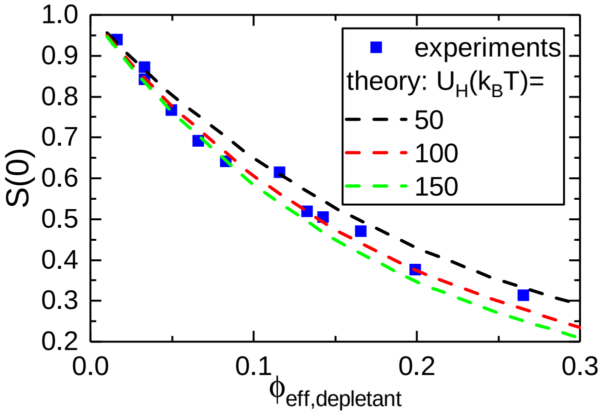

Supplementary Figure 6: $S(0)$ **data for depletants.** Experimental data (symbols) calculated from static light scattering measurements at 15°C are compared to theoretical values based on Hertzian potentials of varying interaction strength (dashed lines).

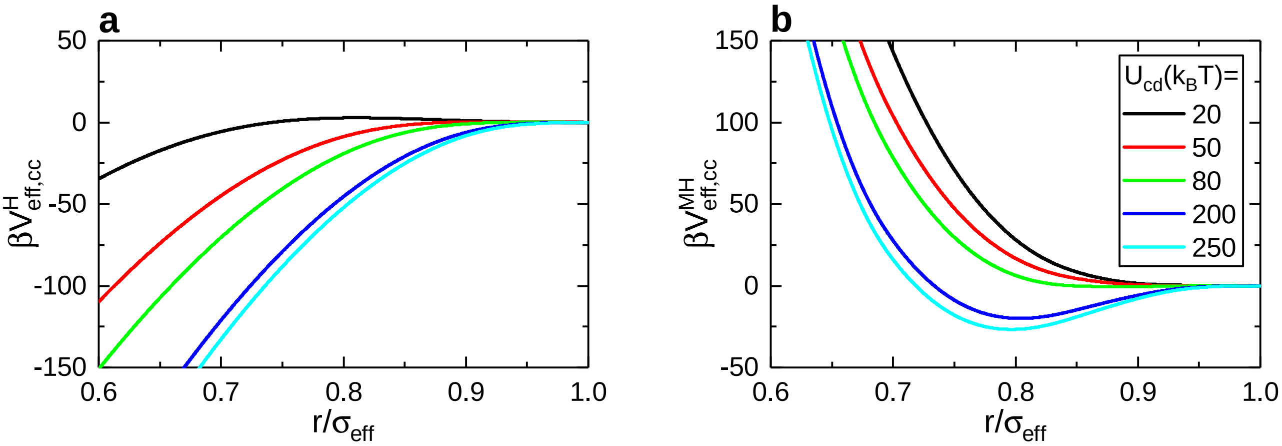

Supplementary Figure 7: Variation of $U_{\mathbf{cd}}$ in Hertzian and multi-Hertzian model. a) Effect of U_{cd} on $V^{\mathrm{H}}_{\mathrm{eff,cc}}$. **b)** Effect of varying U_{cd} on $V^{\mathrm{MH}}_{\mathrm{eff,cc}}$. For the chosen strength (see text) $U_{\mathrm{cd}} = 80k_{\mathrm{B}}T$, the $V^{\mathrm{MH}}_{\mathrm{eff,cc}}$ displays a shallow negative minimum at $\sim -0.6k_{\mathrm{H}}T$. Legend indicates interaction strength and is valid for both panels.

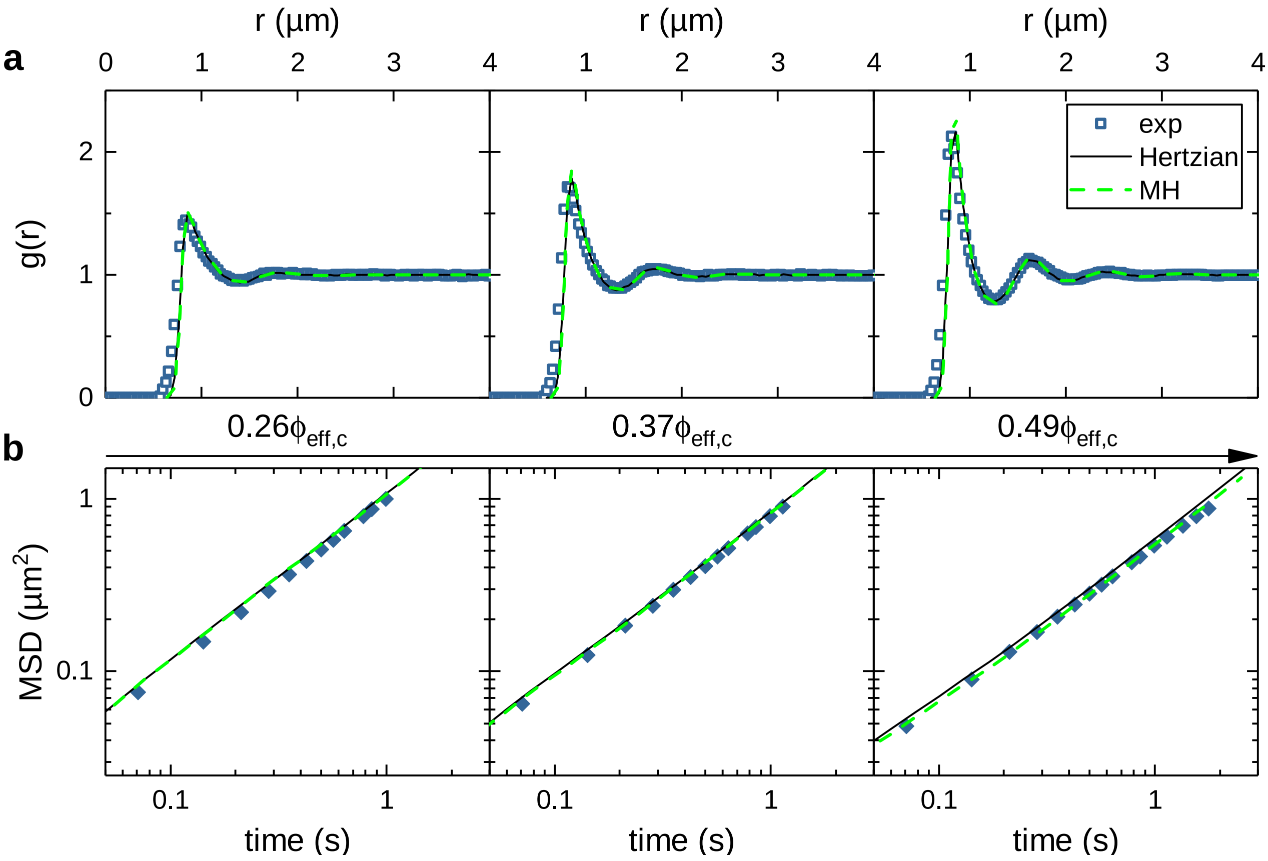

Supplementary Figure 8: Experimental data at 15°C for one-component microgel system overlaid with simulated data from Hertzian and multi-Hertzian model. a) Experimental $g(r)$s are plotted in the colored symbols with $\phi_{\mathrm{eff,c}}$ =0.26, 0.37 and 0.49 (left to right). Black solid lines show the calculated $g(r)$ from simulations based on the Hertzian potential. The overlapping green dotted lines show the calculated $g(r)$ from simulations based on the multi-Hertzian potential. **b)** Identical but for the MSD.

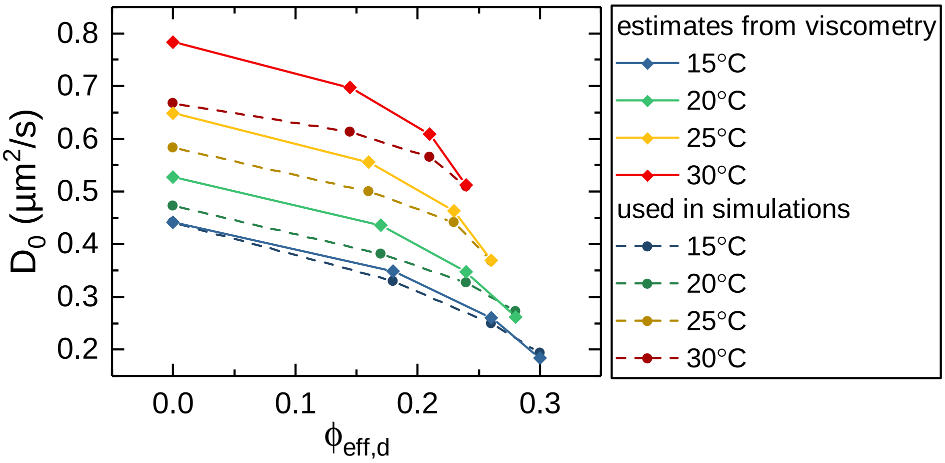

Supplementary Figure 9: Zero-colloid limit self-diffusion coefficients D_0 for each investigated T and $\phi_{\mathrm{eff,d}}$. Symbols connected by solid lines represent the estimates based on experimental data. Symbols connected by dashed lines represent the D_0 values used in simulations, which have been rescaled to $\phi_{\mathrm{eff,d}}^{15^\circ C}$=0.3 for clarity.

Supplementary Notes

Supplementary Note 1: temperature dependent deswelling of the investigated microgels

Supplementary Figure 1 shows the temperature dependent deswelling of the colloid (red symbols) and depletant microgels (black symbols). From this data we directly calculate the change in volume fraction at $T > 15°$C and the size ratio - which is shown on the right axis (green triangles). These parameters are directly used as inputs in the theoretical model.

Supplementary Note 2: bulk viscosity of colloid-only and depletant-only systems

Supplementary Figure 2 shows the relative viscosity for colloid-only samples (panel **a**) and depletant only samples (panel **b**) as function of weight percentage. Data was obtained at 15 and 30°C and fitted with the Batchelor equation. From this, we can obtain a shift factor k (see also Materials and Methods) that allows us to move from wt% to ϕ in the dilute limit. We use this conversion for our initial ϕ_{eff} guesses. In addition, relative viscosity data for the depletant-only samples is used to calculate the theoretical D_0 of colloid particles in the mixtures.

Supplementary Note 3: average deviations between experimental and numerical $g(r)$

Supplementary Figure 3 shows the average deviation between experimental and numerical $g(r)$ defined as $\chi^2 = \sum_i (g_{\mathrm{sim}}(r_i) - g_{\mathrm{exp}}(r_i))^2/g^2_{\mathrm{exp}}(r_i)$. The state points where χ^2 takes the smallest values are the ones at low T and large $\phi_{\mathrm{eff,d}}$. Indeed, in addition to the systematic data noise associated to CLSM, at high T or low depletant packing fractions, the lower spatial resolution in the z-direction and the rapid Brownian motion of the particles lead to a reduction in the peak height and a broadening of the $g(r)$ data at low values of r to the left of the first peak, enhancing the average deviation.

Supplementary Note 4: comparison between the experimental and numerical self-diffusion coefficients

We extracted the diffusion coefficient D from the experimental data with two different approaches. First, we obtain the slope from the two-dimensional MSD versus lag time τ which corresponds to $4D$. This analysis heavily relies on the quality of the MSD and suffers from two main problems: for very short lag times the particle displacement is difficult to resolve, while for very large lag times, the statistics are poor. Therefore, we complement this approach with that adopted in a recent work from Josephson et al. [1]. Hence, we calculate the one-dimensional Van Hove self-correlation for each specific lag time. The Van Hove self-correlation describes the probability of a particle moving Δx in a given τ. With enough statistics, such a probability should follow a Gaussian distribution and thus, the non-gaussian parameter α_2 can be used to describe the shape of the curve. In this way, we pinpoint the first lag time at which α_2 indicates a normal distribution of the Van Hove function, i.e. the first lag time where we get a reliable estimate of the diffusion coefficient with maximal statistics. Only the data at this particular τ is then used to calculate the diffusion coefficient using the Stokes-Einstein relation. The self-diffusion coefficient from simulated data was determined using the slope of the MSD, as the statistics for the simulated MSD is very good up to large lag times.

The data in Supplementary Figure 4 show quite good agreement between experiments and simulations in the range 15-25 °C. For T =30°C the agreement worsens, probably because of the fast diffusion of the microgel particles.

Supplementary Note 5: linear increase of the interaction strengths with temperature

A posteriori, we find that the estimated values for U_{cc} and U_{cd} both increase almost linearly with temperature. The slope of U_{cd} is slightly smaller than that found for U_{cc}, reflecting the fact that the small microgels are slightly softer (because of their lower crosslinker concentration) with respect to the large ones.

Supplementary Note 6: estimation of depletant-depletant interactions

Supplementary Figure 6 shows the measured data for the small wave vector limit $S(0)$ from static light scattering measurements at 15°C for the small depletant particles. $\phi_{\mathrm{eff,d}}$ is calculated using the shift factor k to go from wt% to ϕ. We compare the experimental data points with those calculated for a Hertzian potential using the Rogers-Young closure in the Ornstein-Zernike equation. From the comparison, we find that $U_{\mathrm{dd}} \simeq 100 k_{\mathrm{B}}T$. The exact value is difficult to determine due to the spread in experimental data. However, the exact value is also not so important: whether the interaction strength lies between 50-150 $k_{\mathrm{B}}T$ does not significantly affect our message. Indeed, the key point is that the interactions are very soft, and thus we can assume the depletant interactions to be ideal. In addition, the estimate of U_{dd} can be used to estimate the cross-interactions between colloids and depletants. For additive interactions, any value of U_{dd} would give a much higher value of U_{cd} than what found in our model, suggesting a strong non-additivity of interactions in our soft-soft binary mixture.

Supplementary Note 7: effect U_{cd} on total interaction potential $V_{\mathrm{eff,cc}}$

Supplementary Figure 7 shows the effect of the cross-interaction strength U_{cd} on the total interaction potential $V_{\mathrm{eff,cc}}$. We vary U_{cd} from strongly non-additive at $20k_{\mathrm{B}}T$ to additive at $250k_{\mathrm{B}}T$. We find that even the smallest depletion interaction leads to a fully attractive potential if we use a Hertzian to describe colloid-colloid interactions (panel **a**). If we add depletion attraction to the MH model, we recover a reasonable potential which reflects the stable experimental mixtures (panel **b**).

Supplementary Note 8: comparison of Hertzian and MH model for one-component microgel systems

Supplementary Figure 8 illustrates the comparison between Hertzian (solid lines) and MH (dashed lines) predictions for $g(r)$ (panel **a**) and MSD (panel **b**) of one-component microgel systems. Experimental data (symbols) are also shown as a reference. At low $\phi_{\mathrm{eff,c}} = 0.26$ (left), overlap between the two models is perfect. However, small deviations are evident, particularly in the MSD, for the highest studied volume fraction $\phi_{\mathrm{eff,c}} = 0.49$ (right), where the MH model results are appreciably slower. Indeed, the inclusion of the core repulsion in the MH model is expected to provide a stronger contribution in situations where the microgels are packed tighter or forced together (e.g. due to compression or depletion effects). Thus, even for one-component microgel suspensions, it will be important not to neglect the internal architecture of the microgels to correctly describe the dynamics of the system at high densities.

Supplementary Note 9: comparison of the numerical and experimental zero-colloid limit self-diffusion coefficients

We fix the numerical values of D_0 by comparing the long-time self-diffusion with the experimental data for each investigated T and $\phi_{\mathrm{eff,d}}$. The estimated D_0 values were kept fixed for all studied $\phi_{\mathrm{eff,c}}$. The estimated D_0 are compared to the experimental viscometry values in Supplementary Figure 9, finding a good agreement at large depletant concentrations and low temperatures, where hydrodynamic interactions are less important.

Supplementary References

[1] Josephson, L. L., Furst, E. M. & Galush, W. J. Particle tracking microrheology of protein solutions. *J. Rheol.* **60**, 531–540 (2016).
